# Bitesize Biosecurity: A tool and framework for curating and summarising expert biosecurity advice for farmers using artificial intelligence

**DOI:** 10.1002/vro2.70035

**Published:** 2026-05-22

**Authors:** Alexander F. B. Carmichael, Lorna A. Pate, Andrew J. Duncan, Lynsey Melville, Kate Lamont

**Affiliations:** ^1^ Centre for Epidemiology and Planetary Health Scotland's Rural College Inverness UK; ^2^ Rural Policy Centre Scotland's Rural College Edinburgh UK; ^3^ UHI Inverness University of the Highlands and Islands Inverness UK; ^4^ Moredun Research Institute Pentlands Science Park Edinburgh UK

## Abstract

**Background:**

Biosecurity practices mitigate disease risk on farms; however, adoption and consistent implementation remain variable across farmers. Improved communication of biosecurity information could increase uptake, reduce disease incidence and improve productivity, profitability and animal welfare.

**Methods:**

This project developed a framework leveraging large language models (LLMs) to transform complex biosecurity guidance into accessible information for livestock farmers. The system condenses expert advice from publicly available UK government and veterinary advisory sources to generate more accessible summaries on a purpose‐built platform. Initial implementation targets three UK livestock health challenges for cattle and sheep farmers with the purpose of demonstrating a proof of concept: sheep scab, liver fluke and Johne's disease.

**Results:**

Bitesize Biosecurity provides a centralised repository where farmers can access evidence‐based recommendations without navigating complex literature or multiple websites. The system maintains scientific integrity while presenting guidance in practical, actionable formats.

**Limitations:**

The effectiveness of the framework depends on the quality and availability of source materials. In this study, implementation was limited to three diseases and the UK context, with system performance reliant on LLM accuracy in content transformation process and summary generation.

**Conclusion:**

This tool addresses critical gaps in agricultural communication by streamlining knowledge transfer between research institutions and practitioners. The scalable architecture enables expansion to additional diseases, farm types and regions, with the potential to transform biosecurity information communication across the agricultural sector.

## INTRODUCTION

Good biosecurity practices can mitigate the risk of disease entering and spreading on‐farm; however, implementation of these measures is often inconsistent.^1^, ^2^ Previous research has found that disease epidemics in livestock can negatively impact farmers’ mental health and social wellbeing, including feelings, daily activities and social activities.^3^ Improving how biosecurity information is communicated to farmers could increase the uptake of recommended practices and thus reduce disease incidence and improve productivity, human and animal welfare and farm profitability.

Livestock disease is estimated to cost the UK livestock sector up to £710 million per year.^4^ The costs of some specific diseases have been estimated, including sheep scab, at up to £202 million per year for the Great Britain sheep industry or £1000‒£2100 for a flock of 30 ewes.^5^ Liver fluke has been estimated to cost the UK sheep industry more than £93 million per year in lost production.^6^ Disease‐related economic losses are also substantial in cattle. Although the cost of Johne's disease is difficult to estimate accurately because of subclinical infection, its economic welfare losses have been reported to be comparable to those of other endemic cattle diseases.^7^ These economic estimates do not consider the negative impacts that animal disease has on the reputation of farmer, the agricultural industry as a whole or on human and animal welfare.

Access to biosecurity information is a key factor influencing farmer behaviour regarding biosecurity and disease prevention.^2^ Farmers are more likely to act on information that they consider is from a ‘trusted broker’.^8^ Gunn et al.^9^ found that differing understandings of biosecurity among farmers and veterinarians, and perceptions of poor stakeholder role clarity, created barriers to implementation. The main sources of biosecurity information for farmers are their veterinarians.^10^ The health planning process acts as an opportunity where farmers may talk to their veterinarians about biosecurity; however, uptake in active health planning has been suggested to be modest.^11^

Despite several studies highlighting the importance of the farm veterinarians in conveying information about animal disease and biosecurity,^9^, ^12^ some suggest that this could be changing.^13^, ^14^ There may be an opportunity to create new ways to communicate information about biosecurity that would allow farmers to access up‐to‐date recommendations for free via their smartphones. The use of phone apps in the UK is still increasing; they can be relatively cheap and can be used to access information quickly and conveniently, including personalised information that can be provided offline.^15^ These features, especially the ability to tailor information, may help improve the uptake of information among farmers.^8^ First, information could be personalised to factors relating to the farm, streamlining the information presented and reducing mixed messages. Second, the information could be personalised to meet the learning preferences of the user.

There are high levels of neurodiversity within the farming community, estimated in a recent study to be up to double that of the general population, where rates are around 15%.^16^ Neurodiversity is a term that covers a range of conditions, including dyslexia, autism, attention deficit hyperactivity disorder and brain injury. These conditions can result in challenges in communication and social interaction, focus, reading and writing. The ability to tailor outputs could improve uptake of information by users.

This paper explores the creation of a web application that uses artificial intelligence (AI) to summarise existing biosecurity documents written for farmers to create concise and tailored biosecurity advice while addressing some of the barriers previously discussed.

Artificial intelligence is increasingly being applied across veterinary medicine, offering promising solutions to complex information processing challenges.^17^, ^18^, ^19^, ^20^ Large language models (LLMs) are known for their human‐like text generation capabilities,^19^ with applications extending to consultation summarisation^21^ and prescription text analysis.^22^ The dissemination of livestock biosecurity guidance is challenging due to the complexity of technical documents and numerous information sources. In this study, we explored the application of LLMs for knowledge translation and designed a decision support tool to summarise information and present it in an accessible format.

## METHODOLOGY

### Overview

**FIGURE 1:**
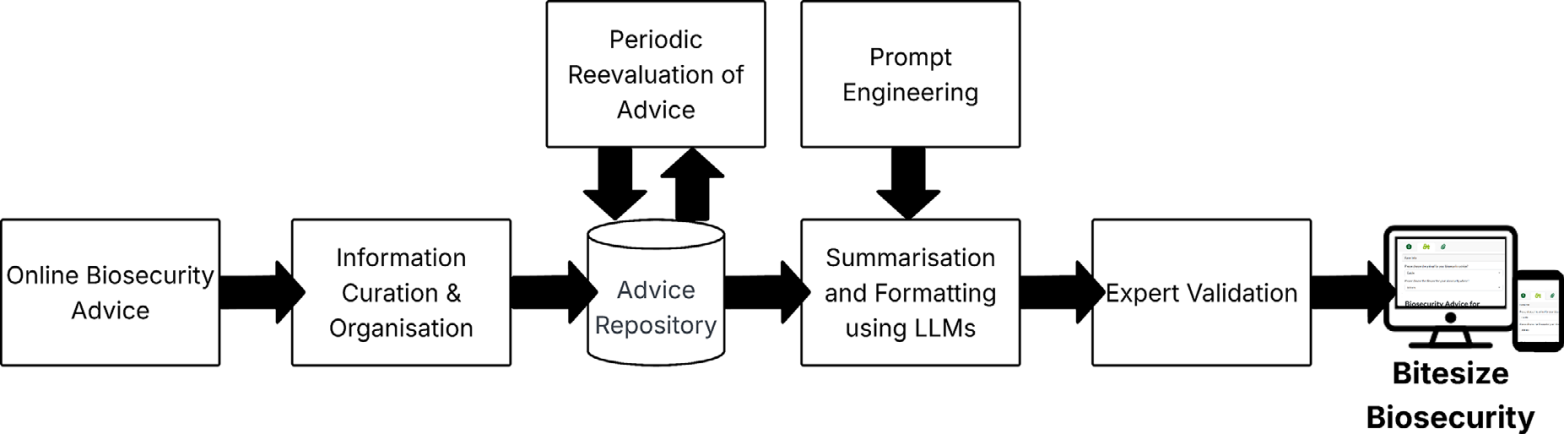
Design framework used when developing Bitesize Biosecurity. Emphasis has been placed on incorporating expert validation and evaluation at multiple steps in the content curation and summarisation process.

The Bitesize Biosecurity framework employs a systematic approach to transform complex biosecurity guidance into more accessible content (Figure 1). The methodology consists of three primary components: advice curation from authoritative sources, prompt engineering to generate accessible content using LLMs, and development of a user interface for effective content delivery. This process maintains scientific accuracy while significantly improving accessibility and practical applicability of available information for the target audience of livestock farmers.

The framework was applied to three significant livestock health challenges in the UK: sheep scab, liver fluke and Johne's disease due to their diverse characteristics, economic impact and ongoing significance within UK cattle and sheep farms.^23^ The methodology was designed to be scalable and adaptable to allow for future expansion to additional livestock diseases and regional contexts.

A limited sample of documents was selected to develop the technological solutions and prompt engineering scripts required. Source materials were obtained from publicly available and authoritative agricultural sources using the following inclusion criteria: relevance to one of the target diseases, practical relevance to farm biosecurity, and origin from government or other established veterinary advisory organisations. The materials were internally validated by four of the authors, whose backgrounds include veterinary epidemiology, livestock health, social science and agricultural research, using a consensus‐based approach and externally validated by a veterinary surgeon with farm animal practice experience. Document selection and validation were conducted during tool development in 2025 through a single‐stage informal consensus review. The tool maintains transparency by listing the sources used and ensuring that generated content undergoes validation to prevent misinformation (see Tables S1–S3). Data are not collected from users, with the platform designed to respect farmer privacy while enabling access to evidence‐based biosecurity guidance. Ethical approval for this study was obtained from SRUC's Social Science Ethics Committee (Ref 196/97613478).

### Information curation and organisation

The advice curation process involved systematic identification and collection of biosecurity guidance from publicly available UK government and veterinary advisory sources. For each target disease, we compiled a manageable sample of 20 documents (reflecting the scale of the project), mainly from UK government‐funded sources, including the Animal and Plant Health Agency (APHA), Agriculture and Horticulture Development Board (AHDB), National Animal Disease Information Service (NADIS) and Moredun Research Institute. The collection of documents included different formats: leaflets, posters, reports and advice sheets. These documents were manually reviewed to ensure that they included key recommendations, protocols and best practices. The curation process revealed significant challenges that justified our approach, including highly technical language barriers, hard‐to‐find documents (i.e., links within large bodies of text and/or requiring multiple clicks to find the correct page on websites) and excessive document length.

Despite these challenges, we would like to highlight the high quality of advice provided in the collated documents. The purpose of Bitesize Biosecurity is not to replace these types of media but to utilise the information they contain effectively for the goal of disseminating engaging biosecurity advice. The curated documents used in this paper can be found in Tables S1–S3.

### Farmer engagement

Farmers at two livestock markets in Scotland (Stirling and Dingwall on 12 and 19 February 2025) were asked for their thoughts about the concept and their input into the design of the tool as part of informal conversations. This approach, recommended by Swain and King^24^ as a pragmatic way to generate data in this type of context, also considers the importance of interviewer positionality.^25^ These conversations involved 16 separate individuals, couples or small groups of farmers. Brief handwritten notes were made during the conversations and later written up, shared with the team, and incorporated into design decisions. No formal demographic data were collected during these informal conversations. These interactions informed design decisions rather than constituting a formal qualitative evaluation.

### Prompt engineering

**FIGURE 2:**
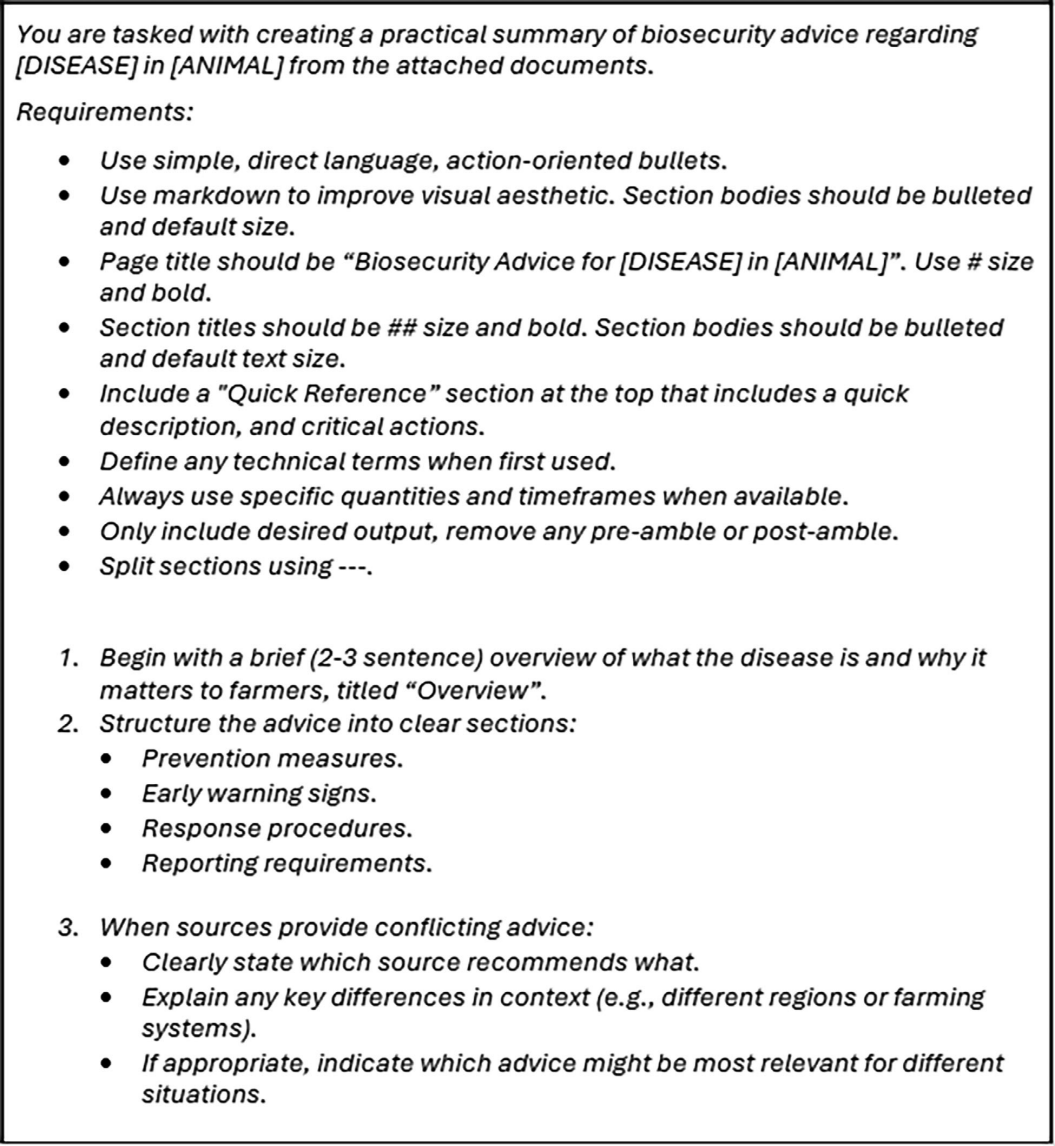
Final prompt following our iterative prompt engineering process. The prompt causes the large language model to generate markdown, which integrates easily onto the Bitesize Biosecurity web application.

Translating complex biosecurity guidance into accessible content required prompt engineering techniques applied to LLMs. Prompt development followed an iterative refinement process, where we improved the prompt based on criteria for the desired output, particularly accuracy and readability. The final prompt is shown in Figure 2. The disease and animal are substituted based on the relevant advice we are looking for. The first section of ‘Requirements’ relates to rules that are applicable throughout the desired output.

A key point to highlight is that we describe the desired markdown format explicitly. Through our iterative prompt engineering process, we found that the stochastic nature of LLMs would become apparent with varying structure between outputs. As it is desirable for Bitesize Biosecurity to have a consistent structure throughout the site, we describe specific rules such as the size and weight of headings, and that sections should be delineated using divider lines. We also explicitly define what section headings should be to encourage a more consistent structure between outputs.

The four key headings were chosen due to their relevance to biosecurity advice across all potential conditions and their ability to quickly let farmers access the actionable advice most relevant to their farm: prevention measures, early warning signs, response procedures and reporting requirements. By using the LLM for this type of formatting, our approach is scalable due to the minimal editing required when reviewing each piece of advice to be published on the tool.

### User interface

For ease of usage in the user interface, the LLM was asked to create a markdown document. These documents were incorporated into a user interface designed using the R statistical software^26^ (version 4.5.1) by creating a Shiny web application (R package version 1.8.1.1).^27^, ^28^ Shiny was chosen due to the speed at which the application could be prototyped due to a pre‐existing server infrastructure already in place, simplifying the release of the application.

**FIGURE 3:**
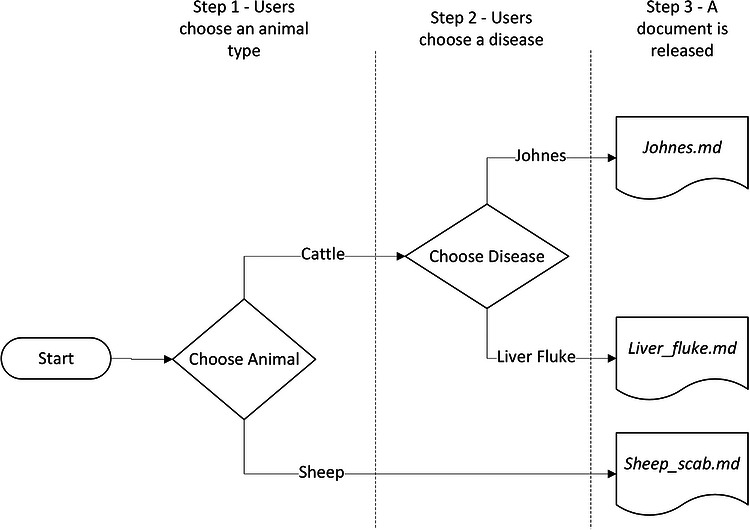
Flowchart representing the choices available to users in the first version of the application.

**FIGURE 4:**
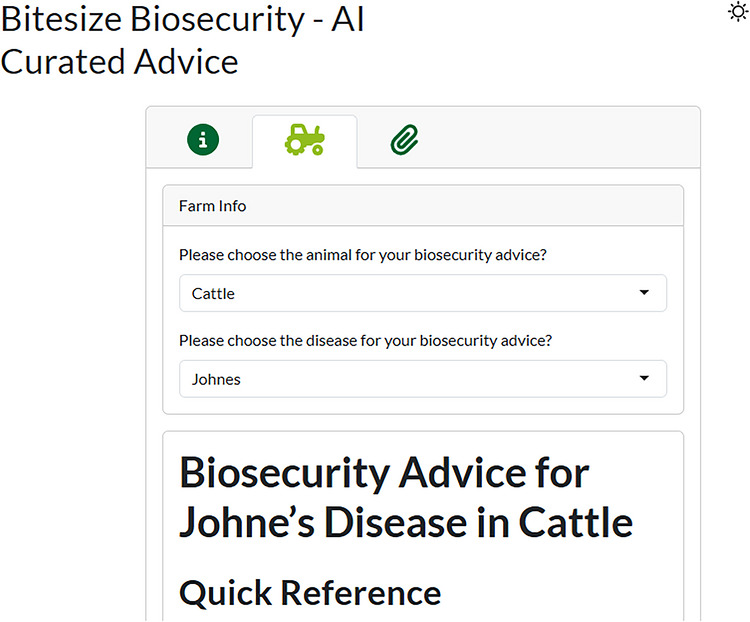
Screenshot of a snippet of the user interface showing the dropdown options for users. AI, artificial intelligence.

In the application itself, users are asked to select from specific choices in several dropdown menus, which then releases the correct LLM markdown file for display. The choices presented to the users, at least those in the first version of the application, are shown in the flowchart in Figure 3. The choices can easily be extended to include additional animal types and diseases. A screenshot of the first version of the user interface for the application is shown in Figure 4.

## RESULTS AND DISCUSSION

The Bitesize Biosecurity framework successfully transformed complex biosecurity guidance into accessible content for three significant livestock health challenges in the UK: sheep scab, liver fluke and Johne's disease. The results presented here relate to knowledge translation and system functionality, not to measured impacts on farmer behaviour or disease outcomes. Future work should evaluate behavioural and on‐farm impacts.

### Advice curation process

The systematic identification and collection of biosecurity guidance revealed several significant challenges that validated our approach. First, the often highly technical language prevalent in expert documents created substantial barriers to comprehension for the average livestock farmer. Documents frequently employed specialised terminology without adequate explanation and assumed background knowledge that many farmers may not possess. Applying this framework beyond the current setting would require adaptation of the curated documents, language and engagement strategies to suit additional contexts.

Second, critical biosecurity information was often difficult to locate, scattered across multiple websites, publications and institutional repositories. This fragmentation created unnecessary obstacles for those seeking comprehensive guidance on specific diseases. Our curation process demonstrated the value of centralised, disease‐specific knowledge repositories that consolidate expert advice from multiple authoritative sources.

Third, traditional biosecurity documents tended to be long, with key actionable recommendations buried within detailed technical content. Some documents exceeded 20 pages, demanding significant time investment from farmers to extract practical guidance; the longest document included in this study was 27 pages. In contrast, the generated summaries for the three focal diseases ranged from approximately 300 to 400 words in length, illustrating the extent to which the framework condensed source material into concise outputs. This finding aligns with research by Grant et al.,^10^ who identified time constraints as a major barrier to biosecurity implementation among livestock farmers.

Despite these challenges, our curation process confirmed the high quality of advice available from trusted sources such as APHA, AHDB and NADIS. The issue was primarily one of accessibility rather than content quality. However, had we included advice from a wider range of sources, including from outwith the UK, the issue of quality and relevance would require careful consideration. These findings are consistent with previous work showing that biosecurity uptake depends not only on the technical quality of advice, but also on how the advice is communicated and the time required to engage with it.^2^, ^9^ Studies of UK livestock farming have also highlighted the importance of relational and communicative factors, particularly the role of veterinary‒farmer interactions, in shaping whether biosecurity advice is acted upon in practice.^10^, ^13^

### Large language models for knowledge translation

Large language models demonstrated strong capability in synthesising and transforming complex biosecurity guidance. Our development process involved informal testing of multiple LLM types, including GPT‐4,^21^ Gemini^29^ and Claude 3.7,^30^ before ultimately selecting Claude 3.7 for our implementation. These models were selected because they were both widely used at the time of development and readily accessible. Model outputs were assessed through informal reviewer judgement by four of the authors rather than through formal quantitative benchmarking, with attention to consistency of structure, clarity of language and fidelity to the original source material. Although this process was exploratory rather than a formal benchmark, it revealed differences in how models handled the task of biosecurity knowledge translation. In particular, we observed differences in the consistency of outputs across repeated or closely related prompts, despite the stochastic nature of these models, with some showing greater stability in phrasing and interpretation than others. More broadly, this aligns with emerging work on the use of LLMs for information retrieval, which highlights their potential to reorganise complex information for end users while also emphasising the need for careful system design and oversight.^31^ Future work should examine open‐source alternatives and systematically evaluate the trade‐offs between performance and computational cost across different model choices.

While the outputs of LLMs are inherently stochastic in nature, we found Claude to produce the most consistent style of response between iterations based on the framework provided in our prompt. However, consistency of style does not guarantee factual reliability, and a key limitation of this approach is the potential of LLMs to generate hallucinations or subtly distort source material.^32^, ^33^ In our framework, this risk was mitigated by grounding summaries in curated expert documents and reviewing outputs for accuracy to the original sources, but it remains an important consideration for future development and evaluation. Given the incredibly rapid rate at which LLMs are being developed and released, we will need to revisit this decision in the future.

### Prompt engineering

A critical component of our framework is the text used to prompt the LLMs to summarise the advice provided across multiple documents. Our initial attempts involved brief, non‐specific requests of only a few sentences, which resulted in substantial structural and content variability between iterations. Because our application required consistent formatting, we introduced additional constraints to reduce this variability. We therefore incorporated highly specific formatting instructions, formulating our requests in terms of markdown syntax. This structured approach significantly improved output consistency, with markdown's explicit structural markers (headers, lists and sections) providing clear organisational cues that the models reliably followed.

While we employed markdown in this implementation, other structured formats, such as HTML, could achieve similar consistency and would be equally suitable for web application integration. We found that this increased specificity in formatting requirements did not compromise the comprehensiveness or accuracy of the content summaries, maintaining the quality of biosecurity advice while achieving the desired structural uniformity. This observation is consistent with recent work showing that prompt design can materially affect the quality, structure and reliability of knowledge extraction from text, with more explicit prompting often improving task alignment and output consistency.^34^ In this sense, our use of the highly structured prompts was not only a practical design choice for application development but also aligned with the growing methodological literature on prompt engineering for knowledge‐focused tasks. The overall strengths, weaknesses, opportunities and threats related to the tool are summarised in Table S4.

### Future work

In the future, we have several planned enhancements and new features to expand the functionality and accessibility of our framework. First, we will substantially broaden our knowledge base to encompass a comprehensive range of pathologies and conditions relevant to the Scottish agricultural community, including both prevalent diseases such as bluetongue, bovine viral diarrhoea and bovine TB, as well as emerging or less common conditions of biosecurity concern.

To improve field accessibility, we are developing a mobile application that will enable farmers to access biosecurity advice directly while working on their farms. We plan for the mobile platform to incorporate an LLM‐powered conversational interface, allowing users to query the curated information database through natural language questions and receive contextually relevant responses in real‐time field conditions.

We will incorporate version control for the generated summaries, including details on changes to the LLMs used, the prompt employed, expert documents added or removed, and updates to the summarised documents. We also investigate multilingual capabilities through regional dialect recognition and translation services. This functionality could address the diverse linguistic needs of Scotland's agricultural workforce, enabling farm workers to access critical biosecurity information in their native languages, thereby removing potential language barriers to compliance.

Finally, we explore the integration of psychological framing techniques into our advice delivery system. By incorporating evidence‐based behavioural psychology principles, we aim to present biosecurity recommendations in ways that are more likely to motivate behavioural change and improve adherence to recommended practices among agricultural stakeholders.

## CONCLUSION

The Bitesize Biosecurity framework successfully demonstrates how LLMs can transform complex biosecurity guidance into more accessible content while maintaining scientific accuracy. By consolidating scattered expert advice from authoritative sources into a centralised platform, the tool addresses critical barriers to biosecurity implementation that have long challenged the livestock sector.

Our systematic approach to advice curation, prompt engineering and user interface design proved effective in creating consistent, actionable guidance for three significant livestock diseases. The framework's structured methodology—featuring standardised sections for prevention, early warning signs, response procedures and reporting requirements—enables farmers to quickly access relevant information without navigating complex technical literature.

The scalable architecture positions Bitesize Biosecurity for expansion across additional diseases, livestock species and regional contexts. With planned enhancements including mobile applications, multilingual capabilities and behavioural psychology integration, the platform has potential to become a useful resource for evidence‐based farm management decisions.

This research establishes a replicable model for AI‐assisted knowledge translation in agriculture, demonstrating that emerging technologies can effectively bridge the gap between scientific expertise and practical implementation. The framework contributes to improved biosecurity communication that ultimately supports better animal health, farm productivity and sector sustainability.

## AUTHOR CONTRIBUTIONS

*Conceptualisation, methodology, software, writing—original draft and writing—review and editing*: Alexander F.B. Carmichael. *Conceptualisation, software, writing—original draft and supervision*: Andrew J. Duncan. *Conceptualisation, investigation and writing—review and editing*: Kate Lamont. *Investigation, writing—original draft and writing—review and editing*: Lorna A. Pate. *Investigation and writing—review and editing*: Lynsey Melville.

## CONFLICTS OF INTEREST

The authors declare they have no conflicts of interest.

## ETHICS STATEMENT

The authors confirm that the ethical policies of the journal, as noted on the journal's author guidelines page, have been adhered to. Ethical approval for this study was obtained from SRUC's Social Science Ethics Committee (Ref 196/97613478).

## Supporting information



SUPPORTING INFORMATION

## Data Availability

All the data are available within the manuscript and the Supporting Information. The web application is currently available here—epidemiology.sruc.ac.uk/shiny/apps/bitesize‐biosecurity/.
