# Supplementary material for: Bitesize Biosecurity: A tool and framework for curating and summarising expert biosecurity advice for farmers using artificial intelligence
Source: Vet Rec Open. 2026 May 22;13(1):e70035. doi: 10.1002/vro2.70035 (PMC13239855; doi:10.1002/vro2.70035)
Supplement: Supplementary file 1 — SUPPORTING INFORMATION [file VRO2-13-e70035-s001.docx]

Bitesize Biosecurity: A Tool and Framework for Curating and Summarising Expert Biosecurity Advice for Farmers using Artificial Intelligence – Supplementary Information

Alexander F. B. Carmichael^1*^, Lorna A. Pate^2^, Andrew J. Duncan^3,1^, Lynsey Melville^4^, Kate Lamont^1^

| 1. SRUC Centre for Epidemiology and Planetary Health, Inverness Campus, Inverness, IV2 5NA | 1. SRUC, Rural Policy Centre, Peter Wilson Building, Kings Buildings, West Mains Road, Edinburgh EH9 3JG | 1. UHI Inverness, 1 Inverness Campus, Inverness, IV2 5NA | 1. Moredun Research Institute, Pentlands Science Park, Bush Loan, Penicuik EH26 0PZ |
| --- | --- | --- | --- |

*Table 1: Sources used in LLM for Liver Fluke biosecurity advice.*

| Source | Available At | Link |
| --- | --- | --- |
| Quarantine Treatments for Liver Fluke | SCOPS | <https://www.scops.org.uk/workspace/pdfs/2-3-1-quarantine-treatments-for-liver-fluke.pdf> |
| Biosecurity Checklist for Key Livestock Diseases | Moredun | <https://moredun.org.uk/resources/factsheets/biosecurity-for-key-livestock-diseases-2> |
| Cattle Purchasing Checklist | AHDB | <https://ahdb.org.uk/knowledge-library/biosecurity-advice-and-cattle-purchasing-checklist> |
| COWS guide to managing liver fluke in bought-in cattle | COWS | <https://www.cattleparasites.org.uk/app/uploads/2018/08/COWS-guide-to-managing-liver-fluke-in-brought-in-cattle1.pdf> |
| Roundworm. Sheep scab. Fluke. Effective quarantine and treatments | SCOPS | <https://www.scops.org.uk/workspace/pdfs/effective-quarantine-and-treatments_1_1.pdf> |
| Fluke Risk and Grazing – a guide to good practise | Moredun | <https://moredun.org.uk/resources/factsheets/fluke-risk-and-conservation-gazing-a-guide-to-good-practice> |
| Biosecurity for Key Livestock Diseases News Sheet Vol. 7 No. 19 August 2023 | Moredun | <https://moredun.org.uk/resources/factsheets> |
| Johne’s Disease (Poster) | Moredun | <https://moredun.org.uk/wp-content/uploads/2021/10/Disease-summary-Johnes_disease.pdf> |

*Table 2: Sources used in LLM for Johne's Disease biosecurity advice*

| Source | Available At | Link |
| --- | --- | --- |
| Johne’s Management Options Chart | SRUC | <https://www.sruc.ac.uk/media/8d8d7f08df7e373/testing_and_management_options.pdf> |
| Johne's disease | AHDB | (<https://ahdb.org.uk/johnes-disease>) |
| Managing and Treating Johne's Disease | AHDB | <https://ahdb.org.uk/farm-excellence/bodysgaw-isa/johnes-management-and-treatment> |
| Johne's Disease | Moredun | <https://moredun.org.uk/research/diseases/johnes-disease> |
| Johnes's disease (Paratuberculosis) | NADIS | <https://www.nadis.org.uk/disease-a-z/cattle/johnes-paratuberculosis/> |
| Glenbervie PARABAN meeting Outlines Lessons Learned | QMS | <https://qmscotland.co.uk/news/johnes-control-needs-long-term-strategy-achieves-results-glenbervie-paraban-meeting-outlines> |

*Table 3: Sources used in LLM for Sheep Scab biosecurity advice*

| Source | Available At | Link |
| --- | --- | --- |
| Biosecurity Checklist for Key Livestock Diseases | Moredun | <https://moredun.org.uk/resources/factsheets/biosecurity-for-key-livestock-diseases-2> |
| Roundworm. Sheep scab. Fluke. Effective quarantine and treatments | SCOPS | <https://www.scops.org.uk/workspace/pdfs/effective-quarantine-and-treatments_1_1.pdf> |
| Stop the Spread: Taking Control of Sheep Scab | Moredun | <https://moredun.org.uk/wp-content/uploads/2023/03/Mfns-7.17-protected-final.pdf> |
| Biosecurity for Key Livestock Diseases News Sheet Vol. 7 No. 19 August 2023 | Moredun | <https://moredun.org.uk/resources/factsheets> |
| Quarantine advice for internal and external parasites | SCOPS | <https://www.scops.org.uk/internal-parasites/quarantine-advice-for-internal-and-external-parasites/> |
| Scab – Treatment and quarantine | SCOPS | <https://www.scops.org.uk/external-parasites/scab/treatment-and-quarantine/> |
| Sheep Scab | Moredun | <https://moredun.org.uk/research/diseases/sheep-scab> |

*Table 4: Strengths, weaknesses, opportunities and threats related to Bitesize Biosecurity*

| Strengths | Successfully demonstrates how LLMs can transform complex biosecurity guidance into accessible, farmer-friendly content while maintaining scientific accuracy. |
| --- | --- |
| Weaknesses | Limited sources, diseases, and formats of advice. |
| Opportunities | Further development of the tool aims to incorporate geographic, topographical, seasonal and production system variability. |
| Threats | Challenges relating to sustainability (environmental, post-project funding support). |
